# Macular thickness and vascular density assessment using optical coherence tomography and optical coherence tomography angiography imaging in iron ore mine personnel

**DOI:** 10.1186/s40942-025-00679-0

**Published:** 2025-05-09

**Authors:** Navid Faraji, Seyyed Pouria Tafti, Niloofar Khoshroo, Alireza Khoshrou, Elham Bakhtiari, Saeid Eslami, Nasser Shoeibi, Mohammad Reza Ansari Astaneh, Seyedeh Maryam Hosseini, Majid Abrishami, Hamid Reza Heidarzadeh, Parnian Arjmand, Mojtaba Abrishami

**Affiliations:** 1 Eye Research Center, Mashhad University of Medical Sciences Khatam-al-Anbia Hospital, Razavi Khorasan Province, Qarani Blvd, Mashhad, 9195965919 Iran; 2 Student research committee, Mashhad University of Medical Sciences, Razavi Khorasan Province, Mashhad, Iran; 3 Department Of Medical Informatics, Mashhad University Of Medical Sciences, Razavi Khorasan Province, Mashhad, Iran; 4 Department of Medical Informatics, Amsterdam Public Health, Amsterdam UMC Locatie AMC, Amsterdam, North Holland Netherlands; 5 Pharmaceutical Sciences Research Center, Mashhad University Of Medical Sciences, Razavi Khorasan Province, Mashhad, Iran; 6 Department of ophthalmology, Mississauga Retina Institute, Mississauga, ON Canada; 7 Ocular Oncology Service, Department of Ophthalmology and Visual Sciences, University of Toronto, Toronto, Canada

**Keywords:** Optical coherence tomography (OCT), Optical coherence tomography angiography (OCTA), Macula, Iron ore mine personnel

## Abstract

**Background:**

To assess macular anatomical and vascular parameters in individuals working in iron ore mines using Optical Coherence Tomography (OCT) and Optical Coherence Tomography Angiography (OCTA) imaging to explore potential correlations between this occupational exposure and retinal changes.

**Methods:**

Individuals from the Sangan iron ore mine in Iran were included in a comparative cross-sectional observational study. An age-matched normal control group was selected from healthy participants employed at Mashhad University of Medical Sciences. Following thorough medical evaluations, participants underwent OCT and OCTA imaging. The macular thickness profile, vessel density (VD) of the superficial (SCP) and deep retinal capillary plexus (DCP), and the area of the foveal avascular zone (FAZ) were measured in our cases and compared with age-matched normal controls.

**Results:**

One hundred and one individuals, with an average age of 38.3 ± 5.59 years in the case group and 38.5 ± 5.59 years in the control group, were enrolled in the study. The difference in mean foveal thickness between cases (50.75 ± 9.13) and normal controls (50.38 ± 8.29) was not statistically significant (*p* = 0.758). Similarly, the mean VD in SCP and DCP for the case group (49.08 ± 2.20 and 49.32 ± 2.42, respectively) and the control group (49.45 ± 3.54 and 49.36 ± 3.97) did not show significant differences. Additionally, there were no significant changes (*p*-value > 0.05) in macular thickness and VD in other retinal regions when comparing the case and control groups.

**Conclusion:**

The research did not establish a significant association between occupational exposure in an iron ore mine and retinal structural changes or alterations in macular VD.

## Introduction

Occupational exposures, which refer to environmental factors consistently affecting individuals in the workplace, can significantly contribute to morbidity among the working-age population. The significance of preventive medicine has been firmly established in recent years to mitigate the impact of diseases and lessen mortality and morbidity rates [1, 2].

Macular thickness and vascular density (VD) alterations have been documented in various ophthalmic conditions such as age-related macular degeneration, glaucoma, and diabetic retinopathy. These alterations are also observed in systemic conditions like metabolic syndrome and polycystic ovarian syndrome [3–5]. Non-invasive and cost-effective methods for measuring these parameters are crucial. These include optical coherence tomography (OCT) and OCT angiography (OCTA) to monitor retinal disorders and prevalent diseases. Such practical and non-invasive techniques comprehensively evaluate the thickness of the macula and peripapillary region, as well as the VD and structures of the retina and optic nerve head [6, 7].

Studies have demonstrated that alterations in retinal thickness and VD may precede the onset of clinically observable symptoms [4, 8–10]. Consequently, it is advantageous to proactively assess these parameters to mitigate their impact on ocular health. For instance, studies have demonstrated the potential for macular OCT and OCTA parameters in detecting early retinal neovascularization changes in patients with diabetes mellitus but without diabetic retinopathy [8]. Moreover, these characteristics can provide a comprehensive analysis for early detection and tracking of disease progression in individuals with multiple sclerosis and Alzheimer’s [4, 9, 10].

The impact of microparticles and pollutants on retinal structure is well-documented [11, 12]. However, there is a significant gap in research investigating the impact of occupational exposure on retinal disease. Existing studies have focused on traumatic and chemical damage to the eye, with a focus on common conditions such as dry eye disease. There is a paucity of literature on choroid, macular and optic nerve head (ONH) thickness and perfusion characteristics secondary to occupational exposures [13–18]. Some studies showed that occupational exposures with materials such as lead and coal had significant impact on retinal thickness and vascular characteristics, but there is no study on the impact of occupational exposure to the iron [19, 20].

Iron ore was chosen for this study due to the occupational exposure of mine personnel to particulate matter, including iron dust, which may have implications for ocular health. Chronic exposure to iron particles and dust has the potential to impact various organs, including the eyes, due to the accumulation of fine particles and the resultant oxidative stress. The severity of the damage and the onset of symptoms are contingent upon the intensity and route of exposure [21]. This study utilizes OCT and OCTA to assess macular thickness and VD, aiming to detect potential subclinical retinal changes associated with long-term occupational exposure. This retrospective cross-sectional study evaluates whether working in an iron ore mine is linked to macular thickness and VD alterations in mine personnel. The findings will help determine the ocular risks of prolonged iron dust exposure and guide potential monitoring strategies.

## Methods

### Study participant

The study recruited participants from the Sangan iron ore mine 300 km southeast of Mashhad in Razavi Khorasan Province, Iran. The participants, aged between 18 and 55, were referred for screening. Initially, approximately 400 individuals were interviewed and examined. Each participant’s medical history was carefully assessed to rule out any underlying ocular conditions. Subsequently, the participants underwent a thorough eye examination, including tests for uncorrected visual acuity (UCVA), best corrected visual acuity (BCVA), automated refraction, and measurement of intraocular pressure (IOP) using a pneumatic tonometer. Following these evaluations, participants voluntarily underwent OCT and OCTA imaging.

The control group comprised healthy individuals employed at Mashhad University of Medical Sciences in Mashhad, Razavi Khorasan Province, Iran. These individuals had not been exposed to iron ore mine dust and had no systemic disorders affecting their eyes. They were also recommended to undergo medical screening.

The study excluded patients with documented systemic disorders affecting the eye, such as hypertension, diabetes mellitus, autoimmune diseases, and glaucoma. Additionally, individuals who were actively breastfeeding or pregnant, or had a history of refractive or ocular surgery, eye injuries, retinopathies, or ONH pathologies were not included. Participants with a BCVA worse than 20/20, a spherical refractive error greater than five diopters, a cylindrical refractive error exceeding two diopters, and an IOP above 25 were excluded.

### Imaging technique

OCT and OCTA scans were obtained using the AngioVue device (RTVue XR Avanti, Optovue, Fremont, CA, USA; Software version 2018.0.0.14). The OCTA system used in this study boasts a high A-scan rate of 70,000 scans per second, ensuring high-resolution imaging. Scans were conducted following pupil dilation. The variables were assessed using the following imaging protocols: AngioRetina 3 × 3 mm (for foveal and parafoveal regions), AngioRetina 6 × 6 mm HD scans (for perifoveal regions), and retina 3D protocols. To report the VD values for the fovea and para-fovea, we relied on the results from the 3 × 3 mm scans. For the VD values of the peri-fovea, we utilized the findings from the 6 × 6 mm scans.

All the captured images met high-quality standards, automated quality index measure (range, 1–10), with a minimum score of 7 out of 10. They were meticulously aligned and centered on the fovea. Any images with artifacts, a quality level below 7/10, or a signal strength index (SSI) lower than 50 were excluded from the study. The scans were performed using consistent equipment and by the same operator to ensure uniformity, with imaging sessions scheduled between 8 AM and 2 PM. Additionally, all participants were instructed not to consume caffeinated liquids, smoke, or take over-the-counter drugs.

We used the spectral domain optical coherence tomography (SD-OCT) technique to measure retinal thickness. Retinal thickness was assessed across three distinct layers:

- The inner retinal layer, measured from the internal limiting membrane (ILM) to the inner plexiform layer (IPL).
- The outer retinal layer, measured from the IPL to the retinal pigment epithelium (RPE).
- The entire retinal layer, spanning from the ILM to the RPE.

**Fig. 1:**
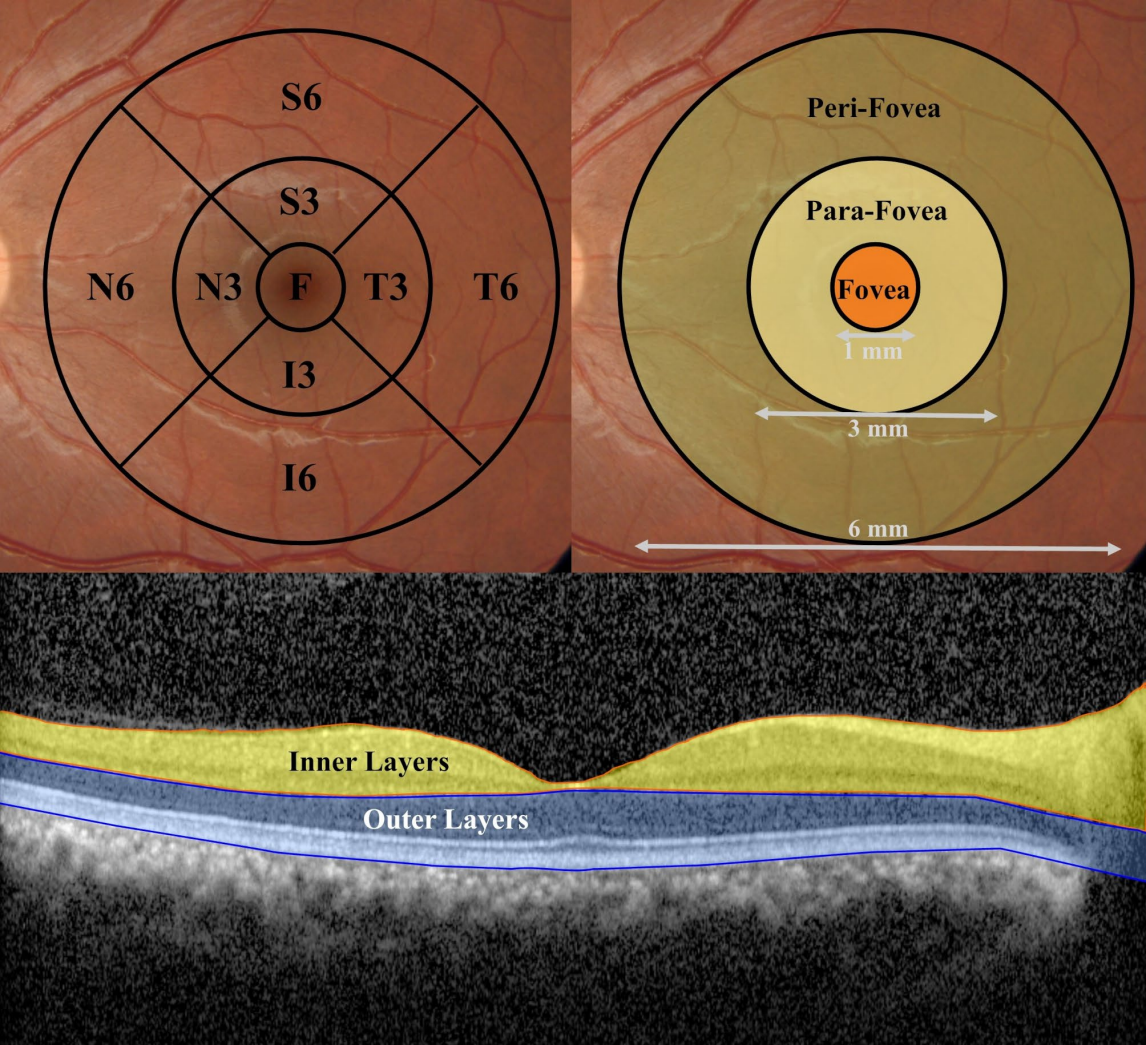
This schematic representation of macular region segmentation was used in Optical Coherence Tomography (OCT) and OCT Angiography (OCTA) analysis. The top Left image shows The Early Treatment Diabetic Retinopathy Study (ETDRS) grid displays the central foveal region (F), the parafoveal quadrants (S3: superior, I3: inferior, T3: temporal, N3: nasal), and the perifoveal quadrants (S6, I6, T6, N6). The top Right image shows the zonal classification of the macula based on the distance from the foveal center: fovea (central 1 mm), parafovea (1–3 mm ring), and perifovea (3–6 mm ring). The bottom image is A cross-sectional OCT B-scan image that illustrates the segmentation of the retina into inner layers (yellow) and outer layers (blue)

**Fig. 2:**
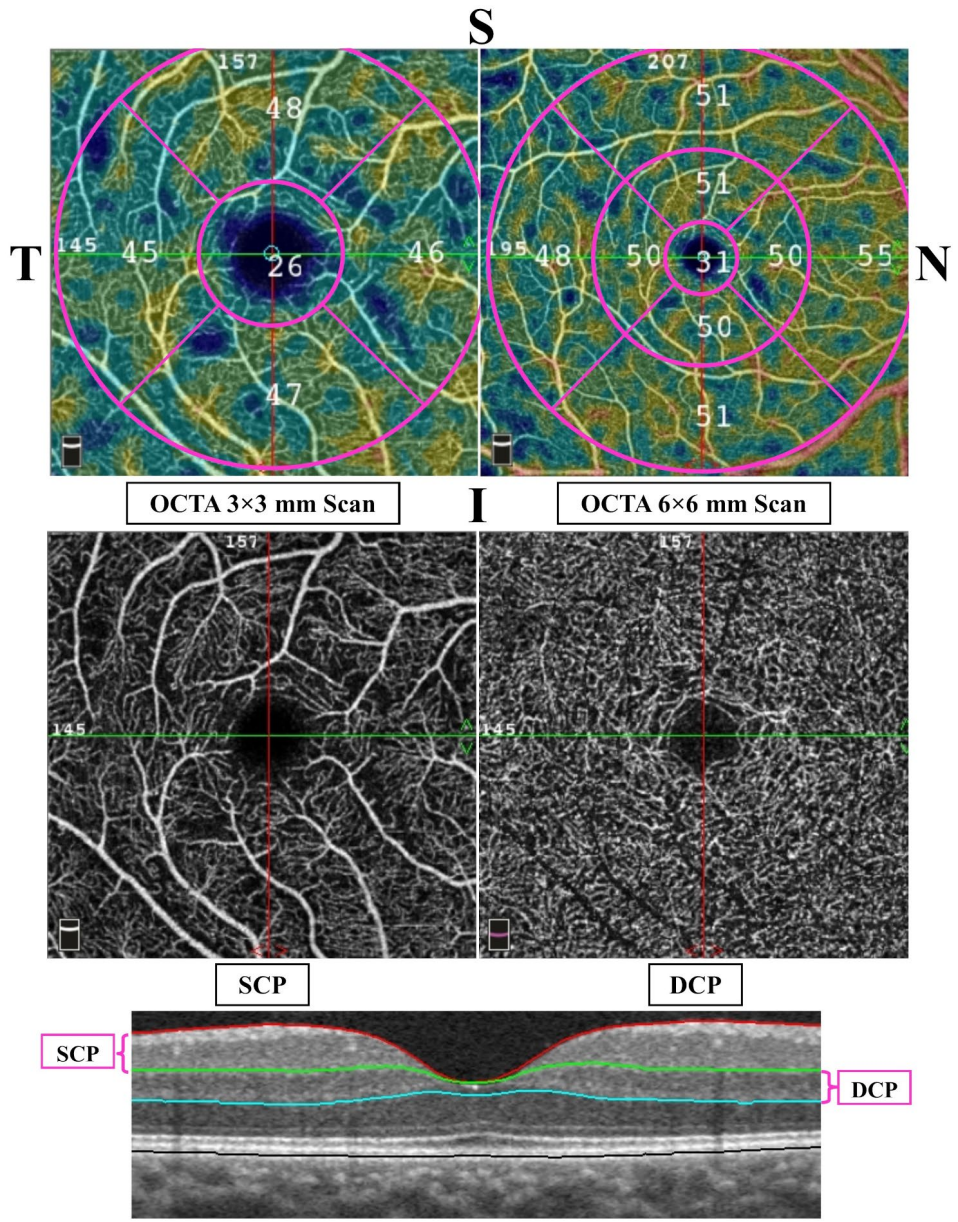
The top images feature enface macular optical coherence tomography angiography (OCTA), showcasing vessel density measurement maps from the Early Treatment Diabetic Retinopathy Study (ETDRS) grid in the superficial inner retina using 3 × 3 mm and 6 × 6 mm protocols. The middle images display enface angiograms of the superficial capillary plexus (SCP) and deep capillary plexus (DCP). The bottom image illustrates segmentation slabs on the B-scan OCT. The SCP is located between the inner limiting membrane and the outer border of the inner plexiform layer, while the DCP is between the outer border of the inner plexiform layer and the outer border of the outer plexiform layer

Scan segmentations were conducted using the Early Treatment Diabetic Retinopathy Study (ETDRS) grid, which includes the central value of a 1 mm diameter ring and four quadrants (superior, inferior, temporal, and nasal) in the inner (3 mm) ring (S3, I3, T3, and N3) and the outer (6 mm) ring (S6, I6, T6, and N6) (Figs. 1 and 2).

### Ethical consideration

The study protocol was conducted according to the principles outlined in the Declaration of Helsinki. Before enrollment, all participants provided informed consent, and the ethical considerations of the study received approval from the Regional Committee on Medical Ethics at Mashhad University of Medical Sciences, located in Mashhad, Iran (IR.MUMS.MEDICAL.REC.1400.847).

### Statistical analysis

The data was analyzed using the Statistical Package for Social Sciences software (SPSS for Mac, version 29.0; IBM SPSS, Inc., Chicago, IL, USA). Descriptive statistics were meticulously calculated, including the mean, standard deviation, median, and range. A paired T-test was used to compare the two eyes’ parameters, revealing no statistically significant differences. Consequently, data from the right eye was exclusively used in subsequent studies.

Furthermore, Levene’s test was employed to assess the homogeneity of variances, and an independent T-test was performed to compare variables between the two groups, with a statistically significant *p*-value set at less than 0.05.

In addition, a control group of 101 individuals was carefully selected, and a propensity score analysis was meticulously conducted to account for any demographic differences between the cases and the control group, enhancing the study’s robustness.

## Results

In the present study, a total of 101 participants were involved. Propensity score matching was utilized to pair participants in the case group with those in the control group. The average age of the patients in the case group was 38.3 ± 5.59, similar to 38.5 ± 5.59 in the control group. The study found an equal proportion of males and females in both groups, with males accounting for 88.1% (89 subjects) and females for 11.9% (12 subjects). The mean scan quality for OCTA in the case group was 8.56 ± 0.57 for the 3 × 3 mm imaging protocol and 8.28 ± 0.59 for the 6 × 6 mm imaging protocol. Meanwhile, in the control group, the mean scan quality was 8.51 ± 0.58 for the 3 × 3 mm imaging protocol and 8.22 ± 0.63 for the 6 × 6 mm imaging protocol.

### Macular thickness

The thickness of the fovea was higher in all three layers of the retina in the case group, with a total increase of 2.115 mm across the entire retinal layer. In the inner retinal layer, all regions’ mean values, except for the N6 and I6, showed a slight rise. The N3 region had the highest increase, measuring 1.443 mm. However, the calculated *p*-value for macular thickness in all regions, including the foveal region and the para- and peri-foveal regions in the inner, outer, and whole retinal layers, was not statistically significant when comparing the case group of 101 individuals with the control group. Nevertheless, there were insignificant changes in the mean values of these areas.

In all areas of the outer retinal layer, except for the fovea, N6, and I6, there was a noticeable decline in thickness. The entire retinal layer’s foveal, N3, and S6 regions exhibited a significant increase in thickness (2.115 mm, 0.935 mm, and 0.172 mm, respectively). In contrast, all other parts of the macula showed a negligible decrease in thickness, as indicated in Table 1.

**Table 1:** Comparison of the macular thickness between groups

		Groups				*P*-Value	Mean difference
		Case		Control			
		Mean	SD	Mean	SD		
Inner Layer	Whole Area	101.404	6.9935	101.250	8.6725	0.889	-0.154
	Fovea	50.759	9.1349	50.380	8.2960	0.758	-0.379
	Para-Fovea	114.513	8.1528	113.764	8.3804	0.521	-0.749
	T3	107.549	7.5294	107.223	7.5655	0.759	-0.326
	S3	117.682	8.7161	116.868	10.1425	0.541	-0.814
	N3	114.493	8.6427	113.050	9.6942	0.265	-1.443
	I3	118.435	9.0138	118.004	8.8927	0.733	-0.431
	Peri-Fovea	99.850	7.3123	99.997	8.4139	0.894	0.147
	T6	87.715	6.2040	87.510	5.9377	0.811	-0.205
	S6	98.791	7.9246	98.193	9.8205	0.634	-0.598
	N6	116.167	9.3591	116.480	11.1498	0.829	0.313
	I6	96.941	7.9139	97.999	9.7109	0.397	1.058
Outer Layer	Whole Area	189.933	8.6957	190.164	8.5301	0.849	0.231
	Fovea	204.561	12.7163	202.826	11.8314	0.316	-1.735
	Para-Fovea	213.374	10.3011	214.052	9.8722	0.633	0.678
	T3	211.094	10.7072	211.838	9.6882	0.605	0.744
	S3	214.636	9.9708	215.525	10.0665	0.529	0.889
	N3	217.452	10.3834	217.961	10.2781	0.727	0.509
	I3	210.450	11.3233	211.032	10.8442	0.710	0.582
	Peri-Fovea	182.085	8.6109	182.239	9.1808	0.903	0.154
	T6	182.138	9.3220	182.484	11.4703	0.814	0.346
	S6	186.126	8.3689	186.551	9.7982	0.740	0.425
	N6	184.520	9.6079	184.384	9.5373	0.920	-0.136
	I6	175.587	9.1308	175.580	8.5731	0.996	-0.007
Whole Retina	Whole Area	291.337	12.7775	291.414	13.7466	0.967	0.077
	Fovea	255.321	19.8737	253.206	17.7106	0.426	-2.115
	Para-Fovea	327.887	15.8661	327.817	14.2885	0.974	-0.07
	T3	318.643	15.8219	319.060	13.6620	0.841	0.417
	S3	332.318	15.8015	332.393	15.8520	0.973	0.075
	N3	331.946	16.2531	331.011	15.7134	0.678	-0.935
	I3	328.885	17.2406	329.036	14.6143	0.947	0.151
	Peri-Fovea	281.935	12.7001	282.236	14.5222	0.876	0.301
	T6	269.852	12.8206	269.994	14.5123	0.941	0.142
	S6	284.917	12.9950	284.745	15.8814	0.933	-0.172
	N6	300.687	15.0160	300.864	16.9955	0.937	0.177
	I6	272.528	12.8834	273.579	14.3253	0.584	1.051

T, Temporal; S, superior; N, nasal; I, inferior; 3, the inner 1–3 mm ring; 6, the outer 3–6 mm ring

The central macular volume slightly increased by 0.00167 mm^3^, while the overall macular volume indicated an unremarkable decrease of 0.00445 mm^3^ (Table 2).

**Table 2:** Comparison of macular volume between groups

	Groups				*P*-Value	Mean difference
	Case		Control			
	Mean	SD	Mean	SD		
Central Region	0.20067	0.015592	0.19900	0.013906	0.422	-0.00167
Whole Macular Volume	8.19880	0.360878	8.20325	0.387506	0.933	0.00445

### Macular vascular density

Of 101 scans, 93 were chosen for angiographic evaluation of macular VD. There were 8 instances of low quality in the OCTA scans. The mean VD for SCP and DCP were 49.0869 ± 2.20380 and 49.3256 ± 2.42219, respectively, for the case group and 49.4580 ± 3.54112 and 49.3634 ± 3.97070 for the control group. The calculated *p*-value shows no significant differences, as reported in Table 4. The comparison of macular VD in each retinal area showed no significant differences between the two groups, with *p*-values greater than 0.05. Nevertheless, there were insignificant alterations in VD in both the SCP and DCP, as depicted in Table 3.

**Table 3:** Comparison of macular vessel density between groups

		Groups				*P*-Value	Mean difference
		Case		Control			
		Mean	SD	Mean	SD		
SCP	Whole Image	49.0869	2.20380	49.3256	2.42219	0.475	0.2387
	S-Hemi	48.8174	2.33172	49.1659	2.36361	0.303	0.3485
	I-Hemi	49.3494	2.24367	49.4794	2.64767	0.714	0.13
	Fovea	19.2367	5.47160	19.1932	5.54084	0.956	-0.0435
	Para-Fovea	49.2692	2.37331	49.1395	2.80962	0.730	-0.1297
	Para-T	47.8075	2.33352	47.5149	2.98371	0.450	-0.2926
	Para-S	50.4568	2.84476	50.4881	3.29521	0.944	0.0313
	Para-N	48.6489	2.52143	48.3300	3.05563	0.431	-0.3189
	Para-I	50.2041	2.84064	50.2529	3.01413	0.908	0.0488
	Peri-Fovea	49.7827	2.33825	50.0034	2.51851	0.529	0.2207
	Peri-T	46.3582	2.44419	46.3469	2.63964	0.976	-0.0113
	Peri-S	48.9338	2.90934	49.4995	2.81962	0.171	0.5657
	Peri-N	53.9227	2.38171	53.9298	2.66300	0.984	0.0071
	Peri-I	49.9623	2.80443	50.2764	3.15681	0.466	0.3141
DCP	Whole Image	49.4580	3.54112	49.3634	3.97070	0.862	-0.0946
	S-Hemi	48.8811	3.46547	49.0270	3.78318	0.780	0.1459
	I-Hemi	50.0103	3.83015	49.6852	4.40742	0.586	-0.3251
	Fovea	35.3867	6.09416	35.8380	6.54504	0.621	0.4513
	Para-Fovea	52.3143	2.80138	52.0629	3.13714	0.558	-0.2514
	Para-T	52.9997	2.61005	52.6093	3.02737	0.339	-0.3904
	Para-S	51.6781	3.19246	51.4449	3.52012	0.630	-0.2332
	Para-N	52.7054	2.77295	52.5211	2.95682	0.656	-0.1843
	Para-I	51.8591	3.41159	51.6657	3.84016	0.712	-0.1934
	Peri-Fovea	50.8309	3.75649	50.6901	4.27895	0.809	-0.1408
	Peri-T	54.8182	3.05805	54.6982	3.42065	0.798	-0.12
	Peri-S	49.0624	4.13380	49.2402	4.51254	0.776	0.1778
	Peri-N	47.8273	4.45827	47.7270	5.26279	0.887	-0.1003
	Peri-I	51.5551	4.53686	51.0380	5.43673	0.475	-0.5171

SCP, superficial retinal capillary plexus; DCP, deep retinal capillary plexus; Hemi, Hemi field; Para, para-fovea; Peri, peri-fovea, VD, vascular density; T, Temporal; S, superior; N, nasal; I, inferior

**Table 4:** Comparison of FAZ characteristics between groups

	Groups				*P*-Value	Mean difference
	Case		Control			
	Mean	SD	Mean	SD		
FAZ Area	0.254	0.09	0.251	0.09	0.770	0.004
PERIM	1.994	0.37	1.976	0.38	0.745	0.018
FD	50.82	3.02	50.66	3.29	0.714	0.167
Acircularity index	1.135	0.050	1.137	0.043	0.755	-0.002

FAZ, fovea avascular zone; PERIM, perimeter circumference of the FAZ; FD, foveal vessel density

The study compared the differences in FAZ characteristics, such as FAZ area, perimeter, circularity index, and foveal vessel density (FD), between the two groups. Our analysis showed no significant increase in all the parameters (*p* > 0.05) (Table 3).

## Discussion

In this detailed observational case-control study, we carefully investigated the macular thickness and VD of 101 healthy participants from the Sangan iron ore mine using advanced OCT and OCTA imaging techniques. Our goal was to compare these findings to a control group to identify any potential differences. After thorough analysis, we found no significant alterations in the macular parameters between the study and the control group. However, we did observe minor differences in several parameters when comparing the two groups, which highlights the potential for further investigation.

OCT and OCTA are advanced, non-invasive imaging techniques that provide rapid, high-resolution images. These technologies are highly valuable for proactive healthcare due to their precision and efficiency [7, 22]. Studies have demonstrated the diagnostic significance of measuring retinal and ONH parameters, prompting extensive research into these characteristics across various ocular diseases. For example, investigations have focused on macular parameters and ONH characteristics in patients with glaucoma and diabetes mellitus [23–25]. Additionally, changes in these parameters have been closely examined in conditions such as retinitis [26]. Notably, systemic diseases that initially do not manifest with ocular symptoms have been shown to impact retinal structure and vascular characteristics as observed in OCT and OCTA imaging [5, 27–30].

Occupational exposure to a variety of environmental factors has also been linked to illnesses affecting multiple organs, including the eyes. Research has established that exposure to microscopic particles can have a detrimental effect on ocular health [11]. Given the high prevalence of eye disorders in working populations, early detection and diagnosis of ocular pathologies are critical. Several studies have explored ocular issues across different professions, highlighting conditions such as dry eye disease, as well as traumatic and chemical injuries commonly seen in various industries [13, 14].

Our research aimed to fill a gap in the literature by investigating the impact of occupational exposure on macular thickness and VD. Although we found no statistically significant association between macular thickness or VD and the specific types of occupational exposure evaluated, subtle changes were noted. These included a slight increase in the thickness of all three retinal layers in the fovea. While these variations were minor, they may hold important implications for understanding and managing occupational health. Additionally, other structural and vascular parameters showed slight changes that varied by region and retinal layer (see Tables 2, 3 and 4). Prior research has indicated that retinal structural alterations can occur before clinical symptoms become apparent, particularly regarding environmental and systemic exposures, specially lead and coal exposures [8–12, 19, 20]. Our study contributes to this limited body of literature by focusing on a specific occupational group. It underscores the potential of OCT and OCTA in identifying early changes. This highlights the importance of ongoing research in this field.

The study’s strengths include a comprehensive analysis of macular thickness and VD across multiple regions and the comparison of interocular differences to enhance statistical accuracy. However, the study also encountered limitations, such as challenges in correlating demographic data with the measured parameters. Prior research has shown that macular thickness and VD are affected by age and gender, with retinal layer thickness decreasing with age and males exhibiting reduced superficial VD in the macular region [31–33]. In another study on 792 healthy participants, they reported the normative values for central retina VDs, and whole image SCP and DCP VDs were found to be 45.9 ± 2.6%, 50.2 ± 3%, respectively [32]. They also reported that these values were significantly higher in females and younger participants [32]. Such factors complicate the assessment of environmental exposures, especially on an individual basis. In our study, this issue was further complicated by the gender imbalance between the case and control groups. We addressed this with likelihood-matching scores [34], but the predominance of male participants reflected the occupational characteristics of the group.

Furthermore, this study did not evaluate ONH structural parameters or VD, both of which are critical for understanding the ocular effects of occupational exposure. Nor did it account for the duration and intensity of exposure, which are key variables that could influence ocular health outcomes. Prolonged occupational exposure, whether direct or indirect, may impact these parameters differently, necessitating further research to fully assess its effects.

In conclusion, this observational study explored the macular structural and vascular parameters in 101 healthy personnel exposed to iron ore mining environments, comparing them with matched healthy controls. While we did not find any statistically significant differences between the two groups, we observed subtle, non-significant trends, including a slight increase in foveal thickness and minor variations in VD within the SCP and DCP across several regions. These findings may indicate early or subclinical retinal responses to occupational exposure. Although the observed changes did not reach statistical significance, their consistency across multiple retinal areas may support the hypothesis that prolonged exposure to industrial particulate matter, such as iron dust, may impact retinal microstructure. Notably, these early retinal changes might remain undetectable through conventional screening methods, reinforcing the importance of high-resolution OCT and OCTA imaging in preventive occupational health assessments. Our results highlight the necessity for longitudinal studies with larger sample sizes and more detailed exposure quantification to understand the ocular effects of such working environments fully. Future research that includes ONH and choroidal analysis and long-term follow-up will be essential in identifying potential cumulative impacts on visual health.

## Data Availability

The data that support the findings of this study are available from the corresponding author upon reasonable request.

## Abbreviations

OCTA: Optical coherence tomography angiography
OCT: Optical coherence tomography
VD: Vessel density
DCP: Deep capillary plexus
FAZ: Foveal avascular zone
SCP: Superficial capillary plexus
ONH: Optic nerve head
UCVA: Uncorrected visual acuity
BCVA: Best corrected visual acuity
IOP: Intraocular pressure
SD-OCT: Spectral domain optical coherence tomography
ILM: Internal limiting membrane
IPL: Inner plexiform layer
RPE: Retinal pigment epithelium
ETDRS: Early Treatment Diabetic Retinopathy Study
FD: Foveal vessel density
